# Bone mineral density and body composition in Australians following plant-based diets vs. regular meat diets

**DOI:** 10.3389/fnut.2024.1411003

**Published:** 2024-06-21

**Authors:** Grace Austin, Jessica J. A. Ferguson, Shaun Eslick, Christopher Oldmeadow, Lisa G. Wood, Manohar L. Garg

**Affiliations:** ^1^School of Biomedical Sciences and Pharmacy, University of Newcastle, Callaghan, NSW, Australia; ^2^Food and Nutrition Research Program, Hunter Medical Research Institute, New Lambton Heights, NSW, Australia; ^3^School of Health Sciences, University of Newcastle, Callaghan, NSW, Australia; ^4^Macquarie Medical School, Macquarie University, Macquarie Park, NSW, Australia; ^5^Clinical Research Design, Information Technology, and Statistical Support Unit, Hunter Medical Research Institute, New Lambton Heights, NSW, Australia

**Keywords:** bone mineral density, body composition, lean mass, fat mass, BMI, waist circumference, plant-based diets, vegetarian

## Abstract

**Background and aims:**

Bone mineral density (BMD) and body composition play an important role in maintaining metabolic health and physical functioning. Plant-based diets (PBDs) are known to be lower in protein and calcium, which can impact BMD and body composition. This study aimed to investigate the relationship between various PBDs compared to regular meat diet and whole-body BMD, body composition, and weight status.

**Methods:**

A cross-sectional study was conducted with adults (*n* = 240) aged 30–75 years, who habitually followed dietary patterns: vegan, lacto-vegetarian, pesco-vegetarian, semi-vegetarian, or regular meat eater (48 per group). Parameters were measured using dual-energy x-ray absorptiometry (DXA), and multivariable regression analyses were used to adjust for lifestyle confounders, socioeconomic factors, and BMI.

**Results:**

After adjustments, whole-body BMD and body composition were not significantly different between those following PBDs and regular meat diets, except for lacto-ovo vegetarians, who had significantly lower lean mass by −1.46 kg (CI: −2.78, −0.13). Moreover, lacto-ovo vegetarians had a significantly lower T-score by −0.41 SD (CI: −0.81, −0.01) compared to regular meat eaters. Waist circumference was significantly lower in individuals adhering to a PBD compared to a regular meat diet: vegans by −4.67 cm (CI: −8.10, −1.24), lacto-ovo vegetarians by −3.92 cm (CI: −6.60, −1.23), pesco-vegetarians by −3.24 cm (CI: −6.09, −0.39), and semi-vegetarians by −5.18 cm (CI: −7.79, −2.57). There were no significant differences in lean mass (%), fat mass (% and total), android/gynoid measures, body weight, or BMI across dietary patterns. All dietary patterns met the recommended dietary intake for calcium and protein, and 25-hydroxy-vitamin D status was comparable across groups.

**Conclusions:**

This cross-sectional study found that adhering to a PBD characterized by varying degrees of dairy and meat restriction is not associated with meaningful changes in BMD or body composition, provided that the dietary patterns are planned appropriately with adequate levels of calcium and protein.

## 1 Introduction

Overweight and obesity are leading health concerns in Australia and across the globe (1), with two-thirds (67%) of Australian adults being overweight or obese, according to the 2017–2018 National Health Survey (1). According to the World Health Organization (WHO), obesity is among the top 10 most preventable health risks (2). Associated comorbidities include coronary heart disease, hypertension, diabetes, osteoarthritis, and certain cancers, all of which cause a significant burden on healthcare systems (3). Maintaining a healthy body composition presents a great challenge for people living in the modern obesogenic environment, which promotes a sedentary lifestyle and energy-dense diets (4). Moreover, osteoporosis and osteopenia significantly influence morbidity and mortality rates globally (5). The International Osteoporosis Foundation estimates that one in three women over the age of 50 years and one in five men will experience osteoporotic fractures in their lifetime (5). The adoption of plant-based diets (PBDs) is gaining traction globally, with almost 12% of the Australian and United Kingdom populations adhering to vegetarian or low-meat diets (6, 7). PBDs are known to be lower in key nutrients such as protein and calcium, which can influence muscle mass and bone development (8). Therefore, it is important that dietary patterns focus on achieving a healthy body composition and bone mineral density (BMD) for optimal metabolic health and physical functioning (9).

Body composition refers to the body's core components: fat, protein, minerals, and water. Thus, it is more informative about physical-related health than weight measures. It is also important to assess predisposition to nutrition-related diseases such as obesity since it quantitatively measures various tissues (10). The gold standard measure of body composition is dual-X-ray absorptiometry (DXA), which quantifies amounts of fat mass, lean mass, total mass, fat-free mass, and relative skeletal muscle mass index (RSMI). Meanwhile, body weight (kg) and body mass index (BMI) do not differentiate between such components (9). In addition, DXA measures bone mineral density (BMD) and calculates T-scores, which are widely used in clinical settings primarily to diagnose osteopenia and osteoporosis and assess an individual's risk of developing osteoporotic fractures (11). A healthy body composition that has a higher percentage of lean tissue and a lower percentage of body fat is proven to reduce the risk of cardiovascular disease (CVD), diabetes, and stroke (12), and optimal bone density can reduce the risk of fractures and susceptibility to bone breakage, known to cause significant morbidity and mortality worldwide (13). Therefore, it is important that dietary interventions focus on preserving lean mass while achieving a healthy percentage of body fat and BMD for optimal metabolic health and physical functioning (14). Assessment of body composition as opposed to body weight and/or BMI enhances the efficacy and specificity of investigating and monitoring the effects of dietary patterns and nutrition interventions on changes in fat mass, muscle mass, and overall bone health, which can better inform public health policy and guidelines (9).

The switch to plant-based dietary patterns is not only an emerging societal trend but a global movement. A generalized definition of PBDs used in research includes low or no intake of animal meat or animal-derived food items, and higher intake of foods from plant origins (15). Several systematic reviews report the substantial health benefits obtained from following a PBD, such as the reduced risk of hypertension, hyperlipidemia, overweight and obesity, obesity-related inflammatory markers, type 2 diabetes, and CVD mortality (15–19). To assist with establishing standardized definitions of PBDs in the Australian context, we will use definitions previously implemented in Australian observational studies (20–22), which originated from studies by Mihrshahi et al. (23) and are aligned with the WHO (24).

While observational studies have demonstrated lower body weight, BMI, and visceral adiposity in individuals following various PBDs compared to those who are regular meat eaters (RMEs) (20), and various PBD interventions have demonstrated significant weight loss effects (25), research investigating their effects on body composition remains scarce. A large randomized controlled trial (RCT) reported that an *ad libitum* vegan dietary pattern with two daily meal replacements led to a significant reduction in body fat and preservation of muscle mass in healthy adults for 10 weeks (26). Notably, meat intake was permitted < 1 per week if deemed necessary by the participant. In addition, a cross-sectional study of vegan Buddhist nuns observed that there was no significant difference in lean muscle mass between vegans and omnivores despite the lower intake of dietary protein in the vegan group (27). In contrast to these findings, a 12-week RCT resistance training program led to greater gains in lean body mass and fat-free mass among an omnivorous diet group compared to a lacto-ovo-vegetarian diet in older men (28). Moreover, a cross-sectional study in healthy women reported that a lacto-ovo-vegetarian diet was significantly associated with lower muscle mass when compared to an omnivorous diet (18 vs. 23 kg, respectively) at the same amount of total protein intake per day (29). Overall, studies on vegan and vegetarian dietary patterns and body composition are conflicting and limited in number, and research surrounding other categories of PBDs, including SVs and PVs, is mostly unexplored.

The impact of PBDs on bone health is still unclear. A systematic review comparing animal vs. plant protein intake in adult women found no significant differences in BMD outcomes (30). A meta-analysis supports these findings, with results indicating no clinically significant associations between vegetarians and lower whole-body BMD, despite the overall BMD of vegetarians being 4% lower than that of omnivores (31). Moreover, findings from a meta-analysis reported that vegetarians and vegans had a lower whole-body BMD, and vegans also had higher fracture rates compared to omnivores in individuals < 50 years (32). Studies investigating vegan and vegetarian diets and bone health have been well-documented, with most evidence showing no significant difference in BMD; however, these studies are 6–15 years old and have not explored other subcategories of PBDs, specifically in today's climate. Given the importance of maintaining lean muscle mass and BMD for metabolic health and physical functioning and the growing global adoption of PBDs and availability of plant-based animal alternative food products, it is of value to understand the relationship between plant-based eating, bone health, and body composition (33).

Nutritional composition can influence body composition and bone mineral density. While PBDs typically have lower protein and calcium intake levels than RMEs, research shows that these levels still meet recommended requirements (34). Animal-based proteins are generally considered complete protein sources for supporting indispensable amino acid requirements, while some plant-based proteins may have below-optimal indispensable amino acid provisions (35). In addition, plant-based proteins are less digestible than animal proteins due to their more complex structures and the presence of inhibitors (36). Without adequate protein and calcium intake, which are essential for muscle and bone development, there is a higher risk of osteoporosis and sarcopenia (37). Further research, which evaluates the qualitative and quantitative nutritional profiles of various PBDs, is warranted to comprehensively evaluate the relationship between PBDs, BMD and body composition.

This study aimed to investigate the association between various PBDs compared to regular meat diets and body composition, weight status, and whole-body BMD. The study provides novel population-based evidence from a purposefully recruited sample of individuals already following PBDs, highlighting the potential risks and benefits related to body composition and bone health.

## 2 Methods

### 2.1 Study design and participants

A detailed description of the study method has been previously published (38). In brief, this cross-sectional study was undertaken at the University of Newcastle, School of Biomedical Sciences and Pharmacy, Nutraceuticals Research Program, Callaghan, NSW, Australia. Data were collected from November 2021 to March 2023. Eligible adults were 30–75 years old, following the same dietary pattern for ≥6 months, and had provided written informed consent. They were excluded if they had a current history of diagnosed CVD, were pregnant, were breastfeeding, or had made any significant changes to their dietary pattern or physical activity exercise in the past 6 months. An eligibility screening criteria published elsewhere (38) assessed the weekly consumption of meat, seafood, eggs, and dairy to categorize participants into dietary pattern groups. The recruited participants were habitually consuming one of five dietary patterns (48 = per group): vegan (no animal products), lacto-ovo vegetarian (LOV, no meat, inclusive of eggs ± dairy), pesco-vegetarian (PV, no meat, inclusive of seafood with/without dairy ± eggs), semi-vegetarian (SV, meat consumption ≤ 2 per week), or RMEs (meat consumption ≥7 per week). Individuals who did not fit into the above categories were ineligible to participate.

The participants completed self-reported questionnaires that included medical demographic history, covering age, race, sex, level of education, duration of dietary pattern adherence, smoking status, alcohol intake, prescribed or over-the-counter medications (s), habitual supplement use, occupation, and socioeconomic status. Physical activity level was assessed using a validated self-reported questionnaire, the International Physical Activity Questionnaire (IPAQ, Long Version, October 2002), which was interpreted as the metabolic equivalent of task minutes per week (MET/week) (39). Participants were assessed for diseases known to affect bone mineral density (such as hyperthyroidism, hyperparathyroidism, renal failure, malabsorption, syndrome, alcoholism, inflammatory bowel diseases, multi-myeloma, leukemia, and chronic arthritis) or use of therapies that interfere with bone metabolism (e.g., glucocorticoid, heparin, warfarin, thyroxine, and estrogen). Overweight and obese classifications were based on a BMI of ≥25 kg/m^2^ (40). Obesity based on body fat percentage was defined as >25% body fat for men and >35% for women, which is the WHO reference standard (41). Serum 25-hydroxy-vitamin D [25(OH)D] levels were collected from fasted blood samples (>10 h) on the same day as the study appointment. Mild 25(OH)D deficiency for adults was defined as levels between 25 and 50 nmol/L as per the Australian and New Zealand Bone and Mineral Society, the Endocrine Society of Australia, and Osteoporosis Australia (2005) (42, 43). An optimal or advantageous level for bone health was defined as ≥75 nmol/L as per the Australian Dietary Guidelines (44, 45).

### 2.2 Nutrient intake analyses

The Australian Eating Food Survey (AES^®^) Food Frequency Questionnaire (FFQ) was used to measure the qualitative intake of food groups and food categories. This online, validated, self-administered questionnaire consists of 120 questions and examines food and nutrient intake over the preceding 3–6 months (46, 47). The reported frequency of food intake was consolidated in relation to various food groups, converted to daily equivalents, and reported as serves per day. An accredited practicing dietitian conducted comprehensive 2-day diet histories to measure quantitative nutrient intake that best reflected the current food supply, particularly for plant-based food products. Diet histories collect detailed information on an individual's usual food consumption patterns, including specifics on food products, condiments, spreads, and oils used in preparation methods (48). Since diet histories provide a more accurate assessment of nutrient intake compared to FFQs, they were utilized to quantitatively assess the dietary intake of micronutrients and macronutrients (46). Data were analyzed using version 10 of “FoodWorks” (Xyris^®^, Brisbane, Australia, sourced online).

### 2.3 Body composition and bone density measurements

Body weight (kg), BMI (kg/m^2^), body composition, and bone mineral density were measured in the fasted state (overnight, >10 h) with all clothing removed via dual-energy x-ray absorptiometry (DXA) using the GE Lunar Prodigy DXA machine. All measurements were performed by the same qualified and certified clinicians at the Newcastle Bone Density Center. Body composition measures included fat mass (kg), percentage body fat (%), fat mass index [fat mass/height (ht)^2^], lean muscle mass (kg), percentage lean muscle mass (%), lean muscle mass index (lean mass/ht^2^), android/gynoid fat tissue distribution (% and ratio), and relative skeletal muscle mass (RSMI; kg/m^2^). Bone mineral content (BMC) and whole-body BMD (g/cm^2^) were collected and expressed as T-scores. T-scores represent the number of standard deviations (SD) from the peak bone mass typically observed in individuals aged between 20 and 30 years. As defined by the WHO, osteoporosis is present when whole-body BMD is −2.5 standard deviations (SD) or more below the average value for the young, healthy adult reference population (a T-score of ≤ -2.5 SD). Osteopenia is defined as a T-score that lies between −1 and −2.5 SD (5). A horizontal tensible tape measure positioned on bare skin, midway between the lower rib margin and the iliac crest (approximately in line with the belly button), was used to measure waist circumference (WC) on fasted participants. Two serial measures were taken, and the average of both was used, rounded to the nearest 0.5 cm.

### 2.4 Statistical analyses

Data were assessed for normal distribution via inspection of histograms and quantile plots, and contentious data were expressed as means ± SD or medians (IQR), depending on normality and categorical data as counts (n) and frequencies (%). One-way ANOVA, Kruskal–Wallis, or Fisher's exact tests were used to compare dietary pattern groups. Under the assumption that whole-body BMD between vegans and RMEs was 0.06 g/cm^2^ (a significant difference in clinical relevance) and given that the between-subject standard deviation (SD) on whole-body BMD is 0.10 g/cm^2^, we estimated that a sample size of 45 individuals per dietary pattern group was required to have a power of 80% to detect differences (Cohen's D 0.6 SD) between groups at the confidence interval of 95% (49). Significant variables (*p* < 0.05), along with those deemed important based on expert opinion and literature, were considered potential confounding variables in regression models (50). Since BMI was identified as an important mediator and not a confounder, a seemingly unrelated linear regression model was employed for each outcome, treating dietary patterns as the exposure factor and incorporating the relevant potential confounding variables as covariates (age, sex, physical activity, duration of dietary pattern, height, alcohol intake, calcium and/or vitamin D supplements, and level of education) and BMI as a mediator (50, 51). Bonferroni corrected alpha 0.0008 (*n* = 64 tests) reports the comparison between the *P* < 0.0008 and those where *P* < 0.05 since this is an exploratory study aiming to minimize type 2 errors. Other diagnostic procedures included inspecting residual plots for normality and homogeneity of variance. Whole-body BMD, percentage lean mass, percentage fat mass, and significant variables identified from the regression model were analyzed by various subgroups: sex (men, women), age ( ≤ 50 years, >50 years), and duration of the dietary pattern ( ≤ 10 years, >10 years). Statistical analyses were conducted using StataCorp. 2016 [Stata Statistical Software: Release 17 (College Station, TX, USA: StataCorp LP)].

## 3 Results

### 3.1 Participant characteristics

A total of 240 participants across five dietary patterns (48 per group) were enrolled in the study (Table 1). A little over two-thirds were women, had a mean age of 54 ± 10 years, half were overweight or obese, the majority had a higher education (88%), a small portion were smokers (6%), had comparable high physical activity levels (Mets/week) and height measurements and were following dietary patterns for a mean length of 17 ± years (means ranging from 7–32 years across dietary patterns) (21). Compared to RMEs, vegans were significantly younger, a smaller proportion were retired, and they were following dietary patterns for a shorter duration (21). There were no significant differences across groups for rates of overweight and obesity based on BMI (kg/m^2^) or percentage body fat according to WHO definitions/reference groups (40, 41). Only 7% of the total sample were being treated medically for osteoporosis or osteopenia, which was not significantly different across diet groups. As per WHO definitions using T-score data, 1% of the population had osteoporosis, and 15% had osteopenia (a total of 16%), which was comparable across groups, with PVs having the highest (25%) and vegans having the lowest rates (6%) (5). Eight percent of the sample had a disease known to affect BMD, which was higher in RMEs (9%) compared to individuals adhering to a PBD (1–3%), and hormone replacement therapy use was comparable across groups. Additional participants' characteristics have been reported in more detail elsewhere (21).

**Table 1:** Participant characteristics and biochemical measures across all dietary patterns.

	**Total sample (*n* = 240)**	**Vegan (*n* = 48)**	**Lacto-ovo vegetarian (*n* = 48)**	**Pesco-vegetarian (*n* = 48)**	**Semi-vegetarian (*n* = 48)**	**Regular meat eater (*n* = 48)**	***P***
Women	186 (77.5%)	34 (70.0%)	36 (75.0%)	39 (81.3%)	40 (83.3%)	37 (77.1%)	0.625
Age (years)^a^	53.8 ± 10.3	47.8 ± 10.0^h^	53.7 ± 10.0^i^	55.8 ± 11.0^i^	55.2 ± 8.7^i^	56.5 ± 9.7^i^	< 0.001
BMI (kg/m^2^)	24.4 ± 4.2	24.2 ± 3.9	24.6 ± 4.0	24.1 ± 3.6	24.2 ± 4.4	25.0 ± 5.0	0.873
Overweight/obese^a^^,^ ^b^	100 (41.7%)	17 (35.4%)	23 (48.0%)	20 (41.7%)	16 (33.3%)	24 (50.0%)	0.375
Obese (% body fat)^b^	133 (55.4%)	25 (52.3%)	27 (56.3%)	27 (56.3%)	24 (50.0%)	30 (62.5%)	0.769
Treatment of osteoporosis or osteopenia^c^	16 (6.7%)	0	6 (12.5%)	5 (10.4%)	3 (6.3%)	2 (1.2%)	0.085
T-score for osteoporosis or osteopenia^c^	39 (16.3%)	3 (6.3%)	9 (18.8%)	12 (25%)	7 (14.6%)	8 (16.7%)	0.139
Disease known to affect BMD^d^	19 (7.9%)	2 (4.2%)	5 (10.4%)	3 (6.3%)	3 (6.3%)	6 (12.5%)	0.607
**Medication, vitamin, and mineral supplement use [count (%)]** ^e^							
Vitamin D^a^	73 (30.4)	15 (31.3)	17 (35.4)	19 (39.6)	13 (27.1)	9 (18.8)	0.206
Calcium	35 (14.6)	7 (14.6)	8 (16.7)	7 (14.6)	7 (14.6)	6 (12.5)	0.999
HRT^a^^,^ ^f^	21 (8.8)	2 (4.2)	5 (10.4)	5 (10.4)	4 (8.3)	5 (10.4)	0.724
**Biochemistry**							
25(OH)D (nmol/L)	83.3 ± 28.3	84.3 ± 28.0	79.6 ± 26.9	88.2 ± 30.7	86.9 ± 31.1	77.6 ± 24.1	0.282
≤ 50 25(OH)D (nmol/L) (%)^g^	21 (8.8%)	4 (8.3%)	4 (8.3%)	5 (10.4%)	5 (10.4%)	3 (6.3%)	0.984
≥75 25(OH)D (nmol/L) (%)^g^	142 (59.2%)	31 (64.6%)	27 (56.3%)	30 (62.5%)	30 (62.5%)	24 (50%)	0.608

MET, metabolic equivalent of task minutes; HRT, hormone replacement therapy; 25(OH)D, 25-hydroxy-vitamin D; BMD, bone mineral density; BMI, body-mass index.

The data are reported as means ± SD and counts (percentages) for categorical variables. Contentious data were compared using ANOVA, and categorical data were compared using Fisher's exact.

a This data have been published elsewhere (21).

b Obesity, classified as per body fat percentage, was defined as>25% body fat for men and >35% for women (41).

c T-scores are derived from a dual-energy X-ray absorptiometry scan and defined as ≤ -2.5 for osteoporosis and ≤ -1 to −2.4 for osteopenia. Only 3 (1%) participants had osteoporosis, so the data were combined with osteopenia scores for analyses (5).

d Diseases known to affect BMD included hyperthyroidism, inflammatory bowel disease, coeliac disease, and chronic arthritis. There was no reported use of therapies that interfere with bone metabolism (e.g., glucocorticoid, heparin, and warfarin) except for thyroxine.

e Participants are currently taking medication/supplements as per the medical history questionnaire. Supplements consumed ≥3 times per week were reported. Supplements that included ≤ 3 types of vitamins/minerals were reported separately, and >3 were reported as multivitamins.

f In women only.

g Mild 25(OH)D deficiency for adults was defined as levels between 25 and 50 nmol/L (42). Only one participant fell below 25 nmol/L (24 nmol/L) and was included in the ≤ 50 (nmol/L) analyses. Optimal or advantageous 25(OH)D levels were defined as ≥75 nmol/L (44).

h, i, j Values within the same row without a common superscript letter are significantly different (P < 0.05).

### 3.2 Nutrient intake and vitamin D levels

Individuals adhering to a vegan dietary pattern consumed no animal-based dairy. However, when plant-based dairy alternatives were included in total daily intake of dairy servings, consumption levels became comparable across groups (21) (Table 2). Dietary calcium intake was similar across groups (including fortified dairy alternatives), and all groups met the recommended daily intake of 1,000 mg/day per the Australian Dietary Guidelines (45). Serum 25(OH)D concentrations were comparable across diet groups, and those following PBDs tended to have higher concentrations than RMEs. For each dietary pattern, 10% or less of the sample had a mild vitamin D deficiency ( ≤ 50 nmol/L), 50% or more had an optimal or advantageous vitamin D level (≥75 nmol/L), and there were no significant differences across groups. The use of vitamin D supplements was also comparable across groups, although individuals adhering to a PBD tended to have higher rates than RMEs (25–40 vs. 19%) (21). Additional analyses not included in the results tables showed that 94% of individuals supplementing with vitamin D did not have a deficiency.

**Table 2:** Dietary intake across dietary pattern groups derived from an average of two dietitian-administered diet histories (quantitative) and the AES^®^ FFQ (qualitative data).

**Nutrient/food group serves (per/day)**	**Total sample (*n* = 240)**	**Vegan (*n* = 48)**	**Lacto-ovo vegetarian (*n* = 48)**	**Pesco-vegetarian (*n* = 48)**	**Semi-vegetarian (*n* = 48)**	**Regular meat eater (*n* = 48)**	***P***
**Quantitative data**							
Energy (kJ)^a^	9,957 ± 2,712	9,784 ± 3,254	9,709 ± 3,325	9,013 ± 2,284	9,631 ± 2,501	9,491 ± 2,304	0.506
Protein (%)^a^^,^ ^b^	16.4% ± 4.1	15.4% ± 3.6^f^^,^ ^g^	14.8% ± 4.2^f^	16.8% ± 3.2^g^	15.4% ± 2.8^f^^,^ ^g^	19.8% ± 4.5^h^	< 0.001
Protein (g)	92.1 ± 38.0	90.8 ± 34.3^f^^,^ ^g^	86.1 ± 62.6^f^	87.2 ± 20.9^f^	86.3 ± 23.6^f^	110.0 ± 28.9^g^	0.007
% RDI (g/kg)^c^	173.3% ± 59.9	168.0% ± 58.7^f^	148.5% ± 52.9^f^	168.5% ± 45.1^f^	172.8% ± 55.4^f^	207.5% ± 71.0^g^	< 0.001
Calcium (mg)	1,085 ± 548	1,027 ± 531	1,124 ± 594	1,063 ± 383	1,068 ± 433	1,144 ± 740	0.834
% RDI	108.5%	102.5%	112.4%	106.3%	106.8%	114.4%	0.831
Sodium (mg)	2,345 ± 2,255	2,341 ± 1,118	2,084 ± 836	2,060 ± 784	2,189 ± 941	3,050 ± 4,661	0.167
Potassium (mg)	4,430 ± 2,944	4,645 ± 1,881	4,924 ± 5,500	4,052 ± 964	4,116 ± 1,330	4,410 ± 2,653	0.571
Magnesium (mg)	522.1 ± 233.4	640.1 ± 275.6^f^	553.9 ± 296.7^f^^,^ ^g^	466.3 ± 136.4^g^	502.6 ± 166.9^g^	447.5 ± 205.0^g^	< 0.001
Phosphorus (mg)	1,692 ± 723	1,633 ± 589	1,667 ± 8,856	15,712 ± 381	16,567 ± 437	1,930 ± 1,055	0.131
Iron (mg)	15.0 ± 7.4	19.4 ± 6.4^f^	16.2 ± 12.0^f^^,^ ^g^	13.1 ± 4.0^g^	14.2 ± 4.4^g^	12.3 ± 5.1^g^	< 0.001
Zinc (mg)	11.4 ± 8.4	11.3 ± 4.2	12.7 ± 17.4	9.3 ± 2.5	11.2 ± 3.4	12.6 ± 3.9	0.269
Selenium (μg)	75.7 ± 43.9	67.2 ± 57.1	66.4 ± 45.7	80.4 ± 32.7	79.6 ± 48.0	84.9 ± 27.9	0.114
**Qualitative data**							
Protein-rich foods^a^^,^ ^d^	1.9 ± 0.9	1.5 ± 0.7^f^	1.3 ± 0.5^f^	2.0 ± 0.6^g^	2.0 ± 1.0^f^^,^ ^g^	2.7 ± 0.7^h^	< 0.001
PBMA^e^	0.2 ± 0.6	0.4 ± 0.8^f^	0.3 ± 0.8^f^^,^ ^g^	0.2 ± 0.7^f^^,^ ^g^	0.1 ± 0.1^g^	0.0 ± 1.1^g^	0.008
Meat and PBMA	2.1 ± 1.0	1.9 ± 0.9^f^^,^ ^g^	1.6 ± 0.9^f^	2.2 ± 1.0^g^^,^ ^h^	2.0 ± 1.0^f^^,^ ^g^	2.7 ± 0.7^h^	< 0.001
Dairy^a^	1.6 ± 1.3	0.0 ± 0.0^f^	1.6 ± 1.5^g^	1.8 ± 1.0^g^	1.7 ± 1.3^g^	2.0 ± 1.4^g^	< 0.001
PBDA^e^	1.5 ± 1.9	3.2 ± 2.0^f^	1.7 ± 1.9^g^	1.2 ± 1.9^g^^,^ ^h^	1.2 ± 1.6^g^^,^ ^h^	0.4 ± 1.1^h^	< 0.001
Dairy and PBDA	3.0 ± 1.9	3.2 ± 2.0	3.3 ± 2.2	3.1 ± 1.9	2.9 ± 1.7	2.4 ± 1.7	0.171

AES, Australian Eating Survey^®^ FFQ, Food Frequency Questionnaire; PBDA, plant-based dairy alternative; PBMA, plant-based meat alternatives; RDI, recommended daily intake.

The data are reported as means ± SD and were assessed using ANOVA.

a These data have been published elsewhere (21).

b Presented as a percentage contribution of total energy intake.

c Expressed as a percentage as per national dietary guidelines recommendations. Mean protein (g) intake was expressed as a mean percentage of the recommended intake, which was age and sex-matched: 0.75 g/kg (aged 19–70) and 0.94 g/kg (70+ years) for women and 0.85 g/kg (19–70 years) and 1.07 g/kg (70+ years) for men. Calcium intake (mg) is expressed as a percentage of the recommended RDI of 1,000 mg/day (52).

d Protein-rich foods include meat, poultry, seafood, eggs, legumes, and nuts. The data used to analyze meat exclusion among vegans and LOVs and dairy exclusion among vegans were derived from diet histories.

e PBDA and PBMA intakes were derived from a dietitian administered FFQ.

^f, g, h, i^Values within a row without common superscript letters are significantly different (P < 0.05).

RMEs had a significantly higher protein as a percentage of total energy intake (EN%) by 3–5 EN%, an additional 17–25 g protein per day, and 0.5–1.1 serves of protein-rich foods per day compared to all those consuming a PBD (21) (Table 2). Analyses of protein included the consumption of plant-based meat alternatives. Despite these differences, all groups met the acceptable macronutrient distribution range for protein (15–25 EN%) and grams per kilogram of body weight per the Australian Dietary Guidelines (45). RMEs consumed double the recommended daily intake of grams per kilogram of body weight, and PBDs consumed an average of 50% extra. Energy levels and alcohol intake was comparable across dietary patterns; further details on nutrient intake have been published elsewhere (21).

### 3.3 Bone mineral density

After adjustments, whole-body BMD and BMC were not significantly different between those following PBDs and regular meat diets (Table 3). Other bone health-related outcomes showed that LOVs had a significantly lower T-score by −0.41 SD (−0.81, −0.01, *P* = 0.042) than RMEs. In the unadjusted model, vegans had a significantly higher whole-body BMD than PVs (Supplementary Table 1).

**Table 3:** Adjusted mean differences in bone mineral density, weight status, and body composition of plant-based diets compared to the regular meat read diets derived from DXA scans.

**Comparisons to regular meat eater**	**Vegan (*****n*** = **48)**		**Lacto-ovo vegetarian (*****n*** = **48)**		**Pesco-vegetarian (*****n*** = **48)**		**Semi-vegetarian (*****n*** = **48)**	
	β **(95% CI)**	***P***	β **(95% CI)**	***P***	β **(95% CI)**	***P***	β **(95% CI)**	***P***
**Bone health**								
WB BMD (g/cm^2^)	−0.01 (−0.02, 0.00)	0.233	0.00 (−0.01, 0.00)	0.230	0.00 (−0.01, 0.00)	0.473	0.00 (−0.02, 0.00)	0.267
T-score (SD)	−0.14 (−0.57, 0.28)	0.514	−0.34 (−0.77, 0.09)	0.122	−0.41 (−0.81, −0.01)	**0.042**	−0.05 (−0.48, 0.39)	0.826
BMC (kg)	−0.04 (−0.16, 0.09)	0.577	−0.08 (−0.18, 0.02)	0.119	−0.09 (−0.19, 0.00)	0.057	−0.01 (−0.11, 0.09)	0.854
**Weight status**								
Weight (kg)	−1.64 (−7.12, 3.84)	0.556	−1.10 (−6.13, 3.92)	0.666	−2.55 (−7.44, 2.35)	0.307	−2.30 (−7.45, 2.86)	0.381
WC (cm)	−4.67 (−8.10, −1.24)	**0.008**	−3.92 (−6.60, −1.23)	**0.004**	−3.24 (−6.09, −0.39)	**0.026**	−5.18 (−7.79, −2.57)	**< 0.001** ^*^
BMI (kg/m^2^)	−0.18 (−2.22, 1.87)	0.866	0.02 (−1.86. 1.90)	0.983	−0.53 (−2.36, 1.30)	0.566	−0.54 (−2.47, 1.38)	0.579
**Body composition**								
Lean mass (kg)	−0.77 (−2.58, 1.04)	0.405	−1.46 (−2.78, −0.13)	**0.031**	−1.11 (−2.68, 0.46)	0.165	−0.12 (−1.58, 1.34)	0.875
Lean mass (%)	0.73 (−1.86, 3.31)	0.582	−0.20 (−2.23, 1.82)	0.843	−0.19 (−2.37, 2.00)	0.867	0.91 (−1.18, 2.99)	0.394
Lean mass index (LM/ht^2^)	−0.33 (−0.93, 0.27)	0.279	−0.29 (−0.87, 0.29)	0.323	−0.41 (−0.96, 0.15)	0.153	−0.15 (−0.67, 0.36)	0.558
Fat mass (kg)	−0.64 (−2.73, 1.45)	0.549	−0.09 (−1.87, 1.70)	0.924	−0.01 (−1.97, 1.94)	0.989	−0.84 (−2.79, 1.12)	0.402
Fat mass (%)	−0.41 (−2.94, 2.12)	0.751	0.27 (−1.90, 2.43)	0.810	0.21 (−2.12, 2.54)	0.861	−0.98 (−3.24, 1.28)	0.397
Fat mass index (FM/ht^2^)	−0.20 (−0.98, 0.57)	0.607	0.01 (−0.67, 0.69)	0.975	−0.02 (−0.74, 0.69)	0.951	−0.30 (−1.02, 0.43)	0.424
Android (%)	−2.02 (−6.59, 2.55)	0.387	0.04 (−3.98, 4.07)	0.983	0.06 (−4.17, 4.29)	0.978	−1.88 (−6.11, 2.36)	0.386
Gynoid (%)	0.50 (−2.30, 3.30)	0.725	−0.37 (−3.16, 2.43)	0.796	0.03 (−2.41, 2.47)	0.981	−1.68 (−4.29, 0.94)	0.208
Android/gynoid ratio	−0.05 (−0.15, 0.04)	0.301	0.01 (−0.08, 0.10)	0.821	0.03 (−0.06, 0.12)	0.543	0.00 (−0.08, 0.08)	0.938
RSMI (kg/m^2^)	−0.25 (−0.59, 0.10)	0.162	−0.24 (−0.52, 0.04)	0.090	−0.21 (−0.51, 0.09)	0.167	−0.12 (−0.41, 0.17)	0.416

BMD, bone mineral density; DXA, dual-energy X-ray absorptiometry; WB, whole-body; FM, fat mass; LM, lean mass; RSMI, relative skeletal muscle mass index; SD, standard deviation; BMI, body-mass index.

The data are presented as β coefficients (95% CIs) and p-values. Multivariate regression analyses were used to adjust the model for age (years), sex (women, men), physical activity level (MET/week), duration of dietary pattern (years), height (cm), alcohol intake (g), use of calcium and/or vitamin D supplements (yes, no), level of education (higher education, yes, no), and BMI (kg/m^2^) as a mediator. Bold values indicate statistical significance (P < 0.05). P-values marked with an asterisk meet the Bonferroni-corrected alpha (P = 0.0008).

### 3.4 Body composition

After adjustments, body composition measures were not significantly different between those adhering to PBDs vs. an RME diet, except for lean mass (Table 3). LOVs had significantly lower lean mass by −1.46 kg (CI: −2.78, −0.13, *P* = 0.031) compared to RMEs and vegans, PVs and SVs reported no differences. The remaining body composition parameters, including percentage lean mass, lean mass index, fat mass percentage, fat mass index, android/gynoid (percentages and ratio), and RSMI, were not significantly different in individuals following PBDs compared to RME diets. WC was significantly lower in vegans by −4.67 cm (CI: −8.10, −1.24), LOVs by −3.92 cm (CI: −6.60, −1.23), PVs by −3.24 cm (CI: −6.09, −0.39), −5.18 cm (−7.79, and −2.57) compared to RMEs. Those adhering to a PBD tended to have a lower weight (kg) when compared to RMEs. However, both body weight and BMI were not significantly different across groups. In the unadjusted model, those adhering to a PBD tended to have a higher percentage of lean mass and a lower percentage of fat mass, which was insignificant (Supplementary Table 1).

### 3.5 Subgroup analyses

Whole-body BMD, percentage lean mass, percentage fat mass, and WC were stratified by sex, age, and duration of dietary pattern (Supplementary Table 2). After adjustments and compared to RMEs, women and those >50 years of age retained a significantly lower WC, although men and those ≤ 50 years of age did not, despite having lower measures. Vegans following a dietary pattern for ≤ 10 years had significantly higher lean mass, lower fat mass, and WC when compared to an RME. In addition, LOVs and SVs following a dietary pattern for >10 years (and ≤ 10 years for SVs only) had a significantly lower WC than RMEs.

## 4 Discussion

This cross-sectional study of 240 middle-aged adults found no associations in whole-body BMD and body composition between individuals following PBDs compared to regular meat diets, except for LOVs, which had significantly lower lean mass. Compared to RMEs, WC was significantly lower in individuals adhering to a PBD. However, body weight and BMI were comparable across groups. This study is the first in Australia to purposefully recruit individuals habitually following various PBDs from the community, offering novel population-based evidence on bone health and body composition.

The current study found no significant differences in whole-body BMD between PBDs and regular meat diets. A pooled analysis of 17 cross-sectional studies in mostly women found that, when compared to RMEs, vegans reported significantly lower whole-body BMD by −0.04 g/m^2^ (CI: −0.06 to −0.01; *P* = 0.04) and vegetarians by −0.02 g/m^2^ (CI: 0.04, −0.00), which was borderline statistically significant (53). However, subgroup analyses of the same study revealed no significant differences between PBDs and regular meat diets in those < 50 years old, studies >*n* = 100, and those including both men and women. Another meta-analysis of nine observational studies in mostly women found similar results, reporting that LOVs (including vegans) had a 4% lower BMD in the femoral neck and lumbar spine compared to RMEs. However, the magnitude of the association was clinically insignificant (31). Again, subgroup analyses showed no significant differences in men and a smaller effect in larger studies (~*n* = 800). Notably, these two meta-analyses did not report an assessment of possible confounders. Therefore, the results did not account for the influence of other factors. Moreover, significance was only obtained in women >50 years old. Therefore, the findings may be mainly related to hormonal changes in postmenopausal women (53). A primary cross-sectional study of 210 nuns over 50 years old corroborates these meta-analyses by finding a difference in whole-body BMD of −0.02 g/m^2^ (CI: −0.06, 0.001) between vegans and RMEs, which did not reach statistical significance with adjustments of clinical and lifestyle factors (31). The results from a 1-year randomized controlled trial (*n* = 1,294, mean age 70 years) showed intervention of a Mediterranean-like diet (low meat, inclusive of fish) together with vitamin D3 supplements (10 μg/d) had no effect on BMD (54). Although our results did not demonstrate a significant difference in whole-body BMD, when expressed as a T-score, there was a significant difference between RMEs and LOVs. This aligns with previous literature, which, in summary, documents slightly lower levels of whole-body BMD in vegan and LOV dietary patterns, though the effect is modest and often non-significant. This was the first Australian-based study to investigate whole-body BMD in other subcategories of habitual plant-based dieters, namely PVs and SVs. Data on other populations around the world are scarce, with one of the few being among Europeans in the “EPIC-Oxford” cohort study, which found the risk of hip fracture was highest in vegans, LOVs, and fish eaters (PVs) (51). Further research is warranted to ascertain the relationship between PBDs and BMD, specifically those inclusive of seafood (PVs) and/or small amounts (SV) of animal meats.

Important nutrients for bone mineralization, supplementation use, dietary intake, and biochemical levels of vitamin D, calcium, and protein were explored. When dietary calcium is deficient, bone demineralization can occur to maintain calcium metabolic balance, which can lead to osteoporosis if stores cannot be replenished (55). Supplemental use of vitamin D and calcium did not differ across dietary patterns, and all groups met the national recommended daily intake levels for both protein and calcium (52). Moreover, with the inclusion of plant-based dairy alternatives, all PBDs met the adequate daily intake for dairy and were comparable to regular meat diets (52). This highlights the importance of including plant-based alternatives in the analyses of nutritional adequacy among individuals following PBDs. Although dietary intake of vitamin D could not be evaluated due to the software used, serum 25(OH)D concentrations were also comparable across diet groups. It is important to consider that the bioavailability of calcium from plant foods is lower than that of animal origin and, therefore, may play a different role in calcium utilization and overall subsequent bone health (56). A meta-analysis of 59 RCTs found that increasing animal-based dietary calcium intake (milk, milk powder, dairy, or unspecified sources) raised BMD by 0.6–1.0% at the total hip and body (57). It was concluded that increasing calcium intake from mostly animal-based dietary sources or taking calcium supplements produces small non-progressive increases in BMD, which are unlikely to lead to a clinically significant reduction in fracture risk (57, 58). Food structure and bioavailability of plant-based calcium may influence BMD outcomes. Future studies are warranted to explore the relationship between BMD and consumption of animal-origin vs. plant-origin calcium to help create specific recommendations for individuals following PBDs or excluding animal-based dairy. Moreover, future studies should quantify the average time spent in the sun to evaluate vitamin D sources more accurately.

In the current study, compared to RMEs, those adhering to a PBD did not show significantly different outcomes for body composition, except for lean mass (kg), which was significantly lower in LOVs only. A systematic review of nine cross-sectional and six RCTs quantifying body composition using DXA scans reported no association between PBDs and body composition. While some studies observed higher muscle mass or a reduced risk of sarcopenia, the majority of these findings were non-significant (59). Notably, PBDs were vaguely defined and included a mixture of vegan, LOV, and plant-based protein dietary patterns, making it difficult to distinguish of effects of specific diets. Furthermore, a meta-analysis of 13 RCTs examining the effects of plant-based protein interventions (including vegan and LOVs) periods ranging from 12 weeks to 1 year found a positive effect on muscle mass and fat loss. However, the difference was non-significant when compared to control groups (animal protein +/- exercise or no control) (49, 50). Overall, it could be concluded that the effects of vegan and LOV diets do not extensively moderate markers of lean mass or fat mass. Literature on PVs and SVs and their association with body composition via DXA is scarce. One large cross-sectional study from the UK Biobank found that low meat eaters, fish eaters, vegetarians, and vegans had a lower body fat percentage (men by −4.5% and women by −4.1%) and lean mass (men by −1.6 and women by −3.5 kg) compared with RMEs (60). Observational and interventional studies are warranted to understand the potential influence of various PBDs, including PVs and SVs, on modulating the indices of body composition.

Protein was 3–5 EN% higher in RMEs (20 EN%) compared to individuals following a PBD (15–17 EN%). Our findings are consistent with a similar cross-sectional cohort study, which described vegans, LOVs, and SVs who consumed 13–14 EN% from protein, also aligned with findings reported in the larger “EPIC Oxford” cohort (61, 62). Despite the difference in protein intake between individuals following PBDs and regular meat diets, all groups met the recommended acceptable macronutrient distribution range for protein (60). The inclusion of plant-based meat alternatives increased the daily consumption of protein-rich foods by 10–15% among PBDs. However, this did not significantly influence results, and RMEs reattained significantly higher intakes than all PBDs. The intake of protein expressed as a percentage of the recommended daily intake, grams per kilo of body weight, was significantly higher in RMEs compared to PBDs. RMEs consumed double the recommended protein intake, and those adhering to PBDs consumed an additional average of 50% more. As mentioned previously, the “EPIC-Oxford Study” reported vegans, LOVs, and SVs to consume ~14% protein as a percentage of energy, which translates to 1.04 g/kg body weight, i.e., 70 g/day, well above the recommended daily intake of 46 g/day for women and 64 g/day for men (52, 62). Another large prospective cohort study, “Adventist Health Study-2”, corroborates the results by reporting vegans' average daily consumption of protein as 71 g/day. Although grams per kilo of body weight were not reported, they also exceeded the recommended estimated daily requirements (63). A meta-analysis of 13 RCTs in adults over 60 years of age found that plant-based diet interventions from 12 weeks to 1 year had positive effects on muscle mass, with no differences compared to animal protein control groups (64). This finding highlights that well-planned PBD can provide adequate protein, potentially explaining why the percentage lean mass was comparable between regular meat eaters and those consuming PBDs, except for LOVs, who had a borderline significantly lower total lean mass (60).

It is important to consider the amino acid composition and availability of proteins when discussing protein adequacy (35). It is well-known that plant-based foods have less optimal amino acid distribution profiles than animal-based foods. Essential amino acids, such as lysine, found in food groups such as grains and cereals have lower than optimal proportions for human needs (63). In developed countries, plant-based proteins typically come from a range of sources and, as such, are more likely to meet the recommended intake of all 20 essential amino acids. This is supported by results from the EPIC-Oxford Study, which found that vegans and LOVs exceeded the estimated average requirement per day for lysine (65). It is granted that inadequate intake of the essential amino acid lysine is more likely to occur in vegans who consume protein from cereals and grains only. However, it has been reported that even consuming a plant-based diet with a limited variety of adequate protein intake can be achieved from a high intake of low-protein foods (66). Another factor to consider is the availability of protein, which has been considered lower in PBDs compared to regular meat diets due to the presence of antinutrients such as phytates, saponins, and tannins. These inhibitory factors, although known to be higher, are not specific to plant-based proteins (37). A study found absorption rates of plant-based protein isolates (pea and wheat) to be 89–92% and those of animal origin (milk, eggs) to be 91–85% (67). Indispensable amino acid availability limitations are perhaps more likely to be related to the structure of proteins, given that plant proteins have complex food matrices that contribute to reduced amino acid absorption. An *in vitro* assay found plant-based proteins have lower numbers of simple protein structures (α-helices and β-sheets), which reduced digestibility and the release of fewer peptides relative to real beef (68). The body of evidence does not show a difference large enough to show the risk of insufficient amino acid absorption (63). More research is warranted to explore the bioavailability of amino acids in commonly consumed plant foods.

Our study demonstrated that those who followed a PBD had significantly lower WCs than those following a regular meat diet. Positive associations between PBDs and weight status have been well-established in previous research. A recent meta-analysis reported that the consumption of PBDs (including vegans and LOVs) was associated with significantly lower body weight, BMI, and WC compared to RMEs. Moreover, when investigating vegans independently, they showed a 5% reduction in body weight (25). Other systematic reviews and meta-analyses, mostly RCTs, similarly reported lower BMI and body weight in those following vegan and LOV dietary patterns (69, 70). One of the few studies to include PVs and SVs is the Australian Longitudinal Study on Women's Health, which found that compared to RMEs, body weight, BMI, and WC were significantly lower in PVs and body weight and BMI lower in LOVs, but not in SVs when compared to RMEs (20). The EPIC-Oxford Study found that when compared to RMEs, body weight, BMI, and WC were lower in PVs and SVs, akin to vegan and LOV dietary patterns (51). In contrast, a 6-month RCT of fifty overweight adults demonstrated vegans' weight loss was significantly different compared to RMEs, though not for SVs or PVs (71). Although WC was the only weight status measure to reach significance, previous literature reports positive associations between PBDs and all weight status markers, including body weight and BMI. Further research that examines weight status among various PBDs, including PVs and SVs, is warranted to substantiate the observations reported in this study.

There are several strengths of the current study, the first being that we were able to examine the association between various PBDs on body composition, weight status, and bone health in individuals habitually consuming dietary patterns from the community. Body composition and bone density were measured using gold standard methodologies (DXA scan), and additional weight status measures were also explored, providing a comprehensive summary of physical health parameters. Previous studies have shown dietary patterns to significantly affect whole-body BMD after a period of >12 months (72) and body composition over a period of >3 months (69). Within this study, the participants were following various PBDs for a mean length of 7–17 years. Therefore, most participants followed dietary patterns long enough to have significant effects. In the absence of a recent national health survey and/or current population-specific food composition database, the use of dietitian-administered diet histories enabled data collection and analysis of food products that reflect the current food supply. Nutrient information was extracted from individual food product packaging for products that were not listed in the existing food composition database, enabling a more accurate reflection of the surge in plant-based food products in the current food supply. Although this study presents findings from a secondary analysis, retrospective power calculations demonstrate that the study sample was adequately powered to examine a statistically meaningful difference in whole-body BMD between vegans and RMEs. Finally, several potential covariates were controlled to avoid confounding and appropriately examine the association between PBDs, body composition, and BMD while considering other lifestyle factors. However, this study is not free of limitations. First, with this study being a cross-sectional study, findings need to be interpreted with caution, and no inferences on causation can be employed. Second, dietary vitamin D intake was unable to be evaluated as the software used did not calculate these data. Dietary vitamin D intake may play a role in bone health outcomes. However, as only 10% of vitamin D absorption is from food intake, the potential influence may be minor (73). Third, some of the data collected were self-reported, including medical and demographic questionnaires and FFQs. However, the data collection tools implemented in this study have been validated in Australian populations (57). Finally, considering the modest sample size of this study, larger population-based prospective cohort studies using standardized methods to categorize various PBDs, with appropriate adjustments, are warranted to verify these results. In addition, future studies should include both qualitative and quantitative assessments of dietary intakes and objective biochemical markers for bone health, including 25(OH)D.

This cross-sectional study of 240 middle-aged adults found that whole-body BMD and body composition were not significantly different between those following PBDs and regular meat diets, except for LOVs, who had significantly but slightly lower lean mass. Those adhering to PBDs had lower WC compared to RMEs, although there were no differences in body weight or BMI. Despite dietary protein being lower in those following a PBD than a regular meat diet, all groups met the recommended requirements for both protein and calcium, and serum 25(OH)D levels were comparable across groups. This study provides novel evidence on the bone health and body composition status of Australians who have adopted various PBDs. Overall, the restriction of animal meat and dairy to varying degrees does not seem to significantly modulate bone health or body composition, provided that protein and calcium intakes are adequate. The findings reinforce the developing understanding that PBDs may not adversely influence BMD or body composition, which is important for clinical use and for supporting national dietary guidelines that encourage plant-based eating. Forthcoming, prospective studies with large sample sizes that clearly define and distinguish different types of plant-based eating and include biochemical measures for bone health are encouraged to substantiate these findings on various PBDs.

## Data availability statement

The original contributions presented in the study are included in the article/Supplementary material, further inquiries can be directed to the corresponding author.

## Ethics statement

The studies involving humans were approved by University of Newcastle's Human Research Ethics Committee (HREC 2020-0195). The studies were conducted in accordance with the local legislation and institutional requirements. The participants provided their written informed consent to participate in this study.

## Author contributions

GA: Conceptualization, Data curation, Formal analysis, Investigation, Methodology, Software, Validation, Visualization, Writing – original draft. JF: Conceptualization, Funding acquisition, Investigation, Methodology, Supervision, Validation, Visualization, Writing – review & editing. SE: Data curation, Investigation, Writing – review & editing. CO: Methodology, Writing – review & editing. LW: Writing – review & editing. MG: Conceptualization, Funding acquisition, Methodology, Project administration, Resources, Supervision, Validation, Visualization, Writing – review & editing.
