# Supplementary material for: Bone mineral density and body composition in Australians following plant-based diets vs. regular meat diets
Source: Front Nutr. 2024 Jun 21;11:1411003. doi: 10.3389/fnut.2024.1411003 (PMC11224549; doi:10.3389/fnut.2024.1411003)
Supplement: Supplementary file 1 [file Table_1.docx]

Supplementary Material

**Supplementary Table 1.** Unadjusted mean ± SD in bone mineral density, weight status and body composition of plant-based diets compared to the regular meat-eating dietary pattern derived from DXA scans.

|  | **Total sample (n=240)** | **Vegan  (n=48)** | **Lacto-ovo vegetarian (n=48)** | **Pesco-vegetarian (n=48)** | **Semi-vegetarian (n=48)** | **Regular meat-eater (n=48)** | ***P*** |
| --- | --- | --- | --- | --- | --- | --- | --- |
| **Bone mineral density** |  |  |  |  |  |  |  |
| WB BMD (g/cm^2^) | 1.2 ± 0.1 | 1.2 ± 0.1^a^ | 1.2 ± 0.2^a,b^ | 1.1 ± 0.1^b^ | 1.2 ± 0.1^a,b^ | 1.2 ± 0.1^a,b^ | 0.034 |
| T-score (SD) | 0.6 ± 1.3 | 1.0 ± 1.2^a^ | 0.5 ± 1.5^a,b^ | 0.2 ± 1.2^b^ | 0.6 ± 1.2^a,b^ | 0.7 ± 1.2^a,b^ | 0.037 |
| BMC (kg) | 2.4 ± 0.5 | 2.6 ± 0.5 | 2.4 ± 0.5 | 2.3 ± 0.4 | 2.3 ± 0.5 | 2.4 ± 0.5 | 0.080 |
| **Weight status** |  |  |  |  |  |  |  |
| Weight (kg) | 69.1 ± 13.3 | 70.4 ± 13.5 | 69.9 ± 13.4 | 67.6 ± 11.4 | 66.5 ± 14.4 | 71.3 ± 13.5 | 0.358 |
| BMI (kg/m^2^) | 24.4 ± 4.2 | 24.2 ± 3.9 | 24.6 ± 4.0 | 24.1 ± 3.6 | 24.2 ± 4.4 | 25.0 ± 5.0 | 0.873 |
| WC (cm) | 86.3 ± 11.7 | 85.0 ± 11.6 | 86.3 ± 10.4 | 86.0 ± 10.8 | 83.6 ± 12.8 | 90.4 ± 12.0 | 0.056 |
| **Body composition** |  |  |  |  |  |  |  |
| Lean mass (kg) | 44.1 ± 9.2 | 46.3 ± 10.0 | 44.1 ± 8.9 | 42.6 ± 7.7 | 42.6 ± 9.9 | 44.9 ± 9.0 | 0.226 |
| Lean mass (%) | 64.0 ± 8.6 | 66.2 ± 8.9 | 63.6 ± 8.5 | 63.3 ± 9.3 | 64.2 ± 8.5 | 62.9 ± 7.6 | 0.364 |
| Lean mass index (LM/ht^2^) | 15.6 ± 2.0 | 15.8 ± 2.0 | 15.6 ± 1.9 | 15.2 ± 1.8 | 15.4 ± 2.1 | 15.8 ± 2.3 | 0.415 |
| Fat mass (kg) | 23.0 ± 9.0 | 21.9 ± 9.0 | 23.6 ± 8.9 | 23.1 ± 9.0 | 22.0 ± 9.5 | 24.5 ± 8.5 | 0.590 |
| Fat mass (%) | 33.7 ± 9.1 | 31.6 ± 9.3 | 34.0 ± 9.2 | 34.4 ± 9.9 | 33.4 ± 9.1 | 34.9 ± 8.2 | 0.455 |
| Fat mass index (FM/ht^2^) | 8.2 ± 3.4 | 7.7 ± 3.4 | 8.4 ± 3.3 | 8.3 ± 3.4 | 8.1 ± 3.6 | 8.8 ± 3.2 | 0.617 |
| Android (%) | 34.4 ± 12.0 | 31.4 ± 12.2 | 35.2 ± 12.0 | 35.3 ± 12.5 | 33.7 ± 12.0 | 36.3 ± 11.2 | 0.302 |
| Gynoid (%) | 37.0 ± 10.7 | 35.7 ± 10.5 | 36.7 ± 11.3 | 37.9 ± 11.2 | 36.4 ± 11.0 | 38.5 ± 9.6 | 0.704 |
| Android/gynoid ratio | 0.9 ± 0.3 | 0.9 ± 0.2 | 0.9 ± 0.3 | 0.9 ± 0.3 | 0.9 ± 0.3 | 0.9 ± 0.3 | 0.683 |
| RSMI (kg/m^2^) | 6.8 ± 1.1 | 6.9 ± 1.2 | 6.8 ± 1.0 | 6.6 ± 1.0 | 6.7 ± 1.2 | 7.0 ± 1.3 | 0.469 |

BMD, bone mineral density DXA, dual-energy X-ray absorptiometry; WB; whole-body; FM, fat mass; LM, lean mass; RSMI, relative skeletal muscle mass index; SD, standard deviation. Differences between groups assessed for significance using ANOVA with post hoc pairwise comparisons.

^a,b,^Values within the same row without a common superscript letter are significantly different (*P* < 0.05).

**Supplementary Table 2**. Mean differences and 95% CI in bone mineral density and body composition of plant-based diets compared to regular meat diets by subgroups sex, age, and duration across dietary pattern groups.

|  | **Vegan** | | | **Laco-ovo vegetarian** | | **Pesco-vegetarian** | | | **Semi-vegetarian** |
| --- | --- | --- | --- | --- | --- | --- | --- | --- | --- |
|  | | | | | **Sex: Men (n=54), Women (n=186)** | |  | | |
| **WB BMD** |  | | |  | |  | | |  |
| Men | -0.05 (-0.15, 0.06) | | | -0.08 (-0.18, 0.03) | | -0.09 (-0.20, 0.02) | | | -0.06 (-0.17, 0.06) |
| Women | -0.01 (-0.07, 0.05) | | | -0.03 (-0.08, 0.03) | | -0.03 (-0.07, 0.02) | | | 0.00 (-0.05, 0.04) |
| **% Lean mass** |  | | |  | |  | | |  |
| Men | -2.25 (-6.71, 2.21) | | | -2.53 (-6.09, 1.02) | | -0.41 (-5.44, 4.62) | | | 1.58 (-3.44, 6.61) |
| Women | 2.17 (-1.05, 5.39) | | | 0.81 (-1.73, 3.35) | | 0.37 (-2.15, 2.88) | | | 1.15 (-1.41, 3.70) |
| **% Fat mass** |  | | |  | |  | | |  |
| Men | 2.44 (-2.33, 7.21) | | | 2.78 (-1.06, 6.61) | | 0.70 (-4.57, 5.98) | | | -1.66 (-7.03, 3.71) |
| Women | -1.83 (-4.90, 1.25) | | | -0.81 (-3.53, 1.92) | | -0.43 (-3.13, 2.28) | | | -1.22 (-3.96, 1.53) |
| **Waist Circumference** | | | |  | |  | | |  |
| Men | | -2.70 (-6.28, 0.88) | | -3.45 (-6.95, 0.04) | | -2.29 (-6.24, 1.67) | | | -3.49 (-8.48, 1.50) |
| Women | | -5.22 (-9.84, -0.60)* | | -4.19 (-7.73, -0.66)* | | -3.66 (-7.55, 0.22) | | | -6.26 (-10.05, -2.46)** |
|  | | | | | **Age: ≤50 years (n=83), >50 years (n=157)** | |  | | |
| **BMD** |  | | |  | |  | | |  |
| ≤50 years | -0.06 (-0.16, 0.03) | | | -0.09 (-0.18, 0.00) | | -0.08 (-0.16, 0.00)* | | | -0.03 (-0.12, 0.06) |
| >50 years | -0.01 (-0.07, 0.05) | | | -0.02 (-0.08, 0.03) | | -0.03 (-0.08, 0.01) | | | -0.02 (-0.06, 0.03) |
| **% Lean mass** |  | | |  | |  | | |  |
| ≤50 years | 2.15 (-3.11, 7.41) | | | -0.83 (-5.24, 3.57) | | -1.21 (-5.90, 3.48) | | | 1.57 (-3.49, 6.63) |
| >50 years | -1.12 (-4.15, 1.90) | | | 0.38 (-1.92, 2.67) | | 0.31 (-2.46, 3.08) | | | 0.69 (-1.57, 2.95) |
| **% Fat mass** |  | | |  | |  | | |  |
| ≤50 years | -1.56 (-6.87, 3.75) | | | 0.88 (-3.66, 5.43) | | 1.37 (-3.39, 6.13) | | | -1.91 (-7.03, 3.22) |
| >50 years | 1.10 (-2.11, 4.31) | | | -0.38 (-2.84, 2.09) | | -0.36 (-3.32, 2.59) | | | -0.76 (-3.19, 1.67) |
| **Waist Circumference** | | | |  | |  | | |  |
| ≤50 years | -4.65 (-12.63, 3.32) | | | -4.88 (-11.61, 1.86) | | -2.56 (-9.42, 4.30) | | | -4.14 (-11.72, 3.44) |
| >50 years | -4.05 (-7.85, -0.25)* | | | -3.33 (-6.43, -0.23)* | | -3.61 (-6.90, -0.33)* | | | -5.69 (-8.78, -2.61)** |
|  | | | **Duration of dietary pattern: ≤10 years (n=138), >10 years (n=102)** | | | | |  | |
| **BMD** |  | | |  | |  | | |  |
| ≤10 years | 0.01 (-0.07, 0.10) | | | -0.03 (-0.11, 0.05) | | -0.02 (-0.09, 0.06) | | | 0.01 (-0.07, 0.09) |
| >10 years | -0.04 (-0.13, 0.04) | | | -0.04 (-0.11, 0.04) | | -0.07 (-0.12, -0.01)* | | | -0.03 (-0.09, 0.04) |
| **% Lean mass** |  | | |  | |  | | |  |
| ≤10 years | 2.98 (0.20, 5.76)* | | | 0.16 (-3.07, 3.40) | | 1.32 (-1.16, 3.81) | | | 1.43 (-0.88, 3.74) |
| >10 years | -3.76 (-8.42, 0.91) | | | 0.10 (-3.08, 3.27) | | -0.67 (-4.02, 2.68) | | | 1.29 (-1.88, 4.47) |
| **% Fat mass** |  | | |  | |  | | |  |
| ≤10 years | -2.72 (-5.38, -0.06)* | | | -0.08 (-3.48, 3.32) | | -1.40 (-3.96, 1.16) | | | -1.54 (-4.00, 0.93) |
| >10 years | 3.92 (-0.97, 8.82) | | | -0.12 (-3.51, 3.28) | | 0.67 (-2.93, 4.28) | | | -1.40 (-4.82, 2.01) |
| **Waist Circumference** | | | |  | |  | | |  |
| ≤10 years | -4.98 (-9.40, -0.56)* | | | -3.24 (-7.96, 1.49) | | -3.36 (-8.17, 1.45) | | | -4.32 (-7.93,-0.71)** |
| >10 years | -3.39 (-8.26, 1.47) | | | -4.66 (-8.80, -0.52)* | | -3.65 (-8.00, 0.70) | | | -6.15 (-10.82, -1.47)** |

BMD, bone mineral density; DXA, dual-energy X-ray absorptiometry; WB, whole-body. Data is presented as β coefficients (95% CIs) and *p*-values. Multivariate regression analyses was used to adjust the model for age (years), sex (female, male), physical activity level (MET/week), duration of dietary pattern (years), height (cm), alcohol intake (g), use of calcium and/or vitamin D supplements (yes, no) and level of education (higher education yes, no) and BMI (kg/m^2^) as a mediator.

**P* <0.05, ***P*<0.005
